# The relationship between low and asymmetric handgrip strength and low muscle mass: results of a cross-sectional study on health and aging trends in western China

**DOI:** 10.1186/s12877-024-05199-4

**Published:** 2024-08-02

**Authors:** Sha Huang, Xiaoyan Chen, Huaying Ding, Birong Dong

**Affiliations:** 1 Department of Geriatric, Zigong Affiliated Hospital of Southwest Medical University, Zigong, Sichuan China; 2 West China Hospital, National Clinical Research Center for Geriatrics, Sichuan University, Chengdu, Sichuan Province China

**Keywords:** Low handgrip strength, Asymmetric handgrip strength, Low muscle mass, Older

## Abstract

**Objective:**

The aim was to determine the relationship between low handgrip strength (HGS) only, asymmetric HGS only, and low HGS combined with asymmetric HGS and low muscle mass in the West China Health and Aging Trends Study (WCHAT) data.

**Study design:**

Individuals aged at least 50 years old were included in this cross-sectional study using WCHAT data. Demographic characteristics, such as age, marital status, education level, ethnicity, and drinking and smoking history, as well as chronic diseases, were recorded for all participants. The HGS of both hands was tested three times using a grip dynanometer with the participant in a standing position with arms extended, before recording the maximum value for both hands. The maximum value referred to values < 28 kg and < 18 kg for males and females, respectively. HGS ratios (non-dominant HGS/dominant HGS) of < 0.90 or > 1.10 suggest asymmetric HGS. The subjects were then allocated to the low HGS, asymmetrical HGS, and combined low and asymmetrical HGS (BOTH group) groups, and those with neither low nor asymmetric HGS (the normal group). The InBody 770 instrument was used for the analysis of muscle mass, with low muscle mass defined as a skeletal muscle mass index (SMI) of < 7.0 kg/m^2^ or < 5.7 kg/m^2^ for males and females, respectively. The associations between the different HGS groups and low muscle mass were assessed by logistic regression analysis.

**Results:**

The study included 1748 subjects, of whom 1272 (72.77%) were over the age of 60 years. The numbers of Han, Tibetan, and Qiang were 885 (50.63%), 217 (12.41%), and 579 (33.12%), respectively. A total of 465 individuals (26.60%) were classified as having low muscle mass, while 228 (13.04%), 536 (30.66%), and 125 (7.15%) participants were allocated to the low HGS, asymmetric HGS, and BOTH groups, respectively. The average SMI differed significantly between the normal group and the other groups (normal group vs. asymmetric HGS group vs. low HGS group vs. BOTH group: 6.627 kg/m^2^ vs. 6.633 kg/m^2^ vs. 6.492 kg/m^2^ vs. 5.995 kg/m^2^, respectively, *P* < 0.05). In addition, the prevalence of low muscle mass in the normal, asymmetric HGS, low HGS, and BOTH groups increased sequentially, with significant differences (normal group vs. asymmetric HGS group vs. low HGS group vs. BOTH group: 21.5% vs. 22.4% vs. 39.5% vs. 56%, respectively, *P* = 0.001). Further logistic regression analysis showed that the presence of low HGS (OR = 1.7, 95%CI: 1.203–2.402) and both low and asymmetric HGS (OR = 3.378, 95%CI: 2.173–5.252) were predictive of low muscle mass, with the chance being higher for the latter condition.

**Conclusion:**

The findings suggest that although asymmetrical HGS itself does not increase the chances of low muscle mass. When low HGS and a combination of both features (low HGS combined with asymmetric HGS) is present in subjects, the chance of low muscle mass increases.

## Introduction

In many diseases, low muscle mass, one of the hallmarks of sarcopenia [1], has been linked to poor clinical outcomes. For example, Xi et al. found that in patients with abdominal trauma, low muscle mass was an independent risk factor for the development of complications, as well as length of hospitalization and hospital costs [2]. Similarly, Xiao et al. found that there was a higher chance of postoperative rehospitalization and complications, 30-day mortality, and overall death in patients with colon cancer and low muscle mass [3]. Furthermore, critically ill patients with COVID-19 who had low muscle mass were more likely to spend longer in both the ICU and general hospital [4], while an association was observed by Wang et al. between low muscle mass and short-term complications in elderly patients after gastrointestinal tumor resection [5]. Hand grip strength (HGS) is a simple method for testing muscle strength. The results not only reflect the level of muscle strength in the whole body but are also related to the level of muscle mass [6–9].

HGS can be measured in a simple, economical, and non-invasive way using only a grip dynamometer. Based on the diagnostic criteria for sarcopenia, as stated by the 2019 Asian Working Group on Sarcopenia (AWGS2019), low HGS is defined as < 28 kg and < 18 kg for males and females, respectively [1]. HGS is a marker of the overall strength of muscles and is not only affected by musculoskeletal conditions but also by neurological function and brain health [10]. Therefore, while the measurements do provide reliable health information, the maximal HGS alone is not sufficient to reflect overall muscle function [11, 12]. In these cases, the presence of variation in the assessment of muscle function may help to predict the chance of adverse health outcomes in clinical and research settings [12–15]. For instance, a large difference in HGS between the two hands is termed asymmetric HGS. This is determined by the HGS ratio, calculated from the highest HGS values for the non-dominant and dominant hands, with a value (ratio of non-dominant HGS/dominant HGS) of < 0.90 or > 1.10 referred to as asymmetric HGS [14]. The latter is also a hallmark of impaired muscle strength and can be of prognostic value to assess muscle function, disease, and adverse clinical events in a simple and non-invasive way [12, 14, 15].

To date, there have been no studies that explore the relationship between low muscle mass and either low HGS only, asymmetric HGS only, or low HGS combined with asymmetric HGS. Therefore, the current study used data from the West China Health and Aging Trends Study (WCHAT) to explore the above relationships. It was hypothesized that there is an association between low muscle mass and either low HGS only, asymmetric HGS only, or a combination of low HGS and asymmetric HGS.

## Methods

### Study design and subject recruitment

This cross-sectional study, approved by the Ethical Review Committee of West China Hospital of Sichuan University (committee number 2017(445) and registration number ChiCTR1800018895), was carried out in accordance with the Declaration of Helsinki. Informed consent was also obtained from each participant prior to the study. For details on the method of data collection, readers are invited to refer to the published research of the research group [16]. Specifically, the study was conducted in multiple areas in western Sichuan. Only follow-up data in 2022 were included in this study. The inclusion criteria were consistent with the baseline data in 2018 (50 years old and above, living in the same area for more than 36 months), and included subjects who had completed three assessments of HGS for both hands as well as a BIA examination during the follow-up in 2022 [16]. The exclusion criteria were consistent with those previously reported for baseline data collection (life expectancy less than six months or failure to provide informed consent) [16]. The following data were recorded for each study participant: age, marital status, education level, race, drinking and smoking history, activities of daily living (ADL) score, gait speed, total protein (TP), prealbumin (P-ALB), albumin (ALB), and chronic diseases, which were further divided into cataracts, chronic obstructive pulmonary disease (COPD), osteoarthritis, history of stroke, diabetes, and coronary heart disease (CHD), and hypertension. These data were obtained in part through face-to-face interviews. The interviewers were from the West China School of Clinical Medicine, Sichuan University, and all received 2 days of training on questionnaire data collection. The items involved in the questionnaire include general characteristics, such as age, ethnic, marital status, education level, smoking history, drinking history, chronic diseases etc. In addition, the contents included in the questionnaire are all classic scales, such as ADL [17]. Blood test data were collected, tested, and reported by professional medical staff. Anthropometric and BIA measurements were collected by trained technicians.

### HGS measurement

A grip dynamometer (EH101; Camry, Zhongshan, China) was used to assess the HGS of both hands three times with the participant in the standing position with the arms straight. The maximum HGS value of each hand was recorded for each measurement. As mentioned above, the threshold value for low HGS was 28 kg and 18 kg for males and females, respectively [1]. The highest HGS values for each hand were then used to calculate the HGS ratio (non-dominant hand to the dominant hand), with values < 0.90 or > 1.10 indicative of asymmetric HGS [14]. Based on the results, the subjects were then classified into the following groups: the low-HGS group consisting of those with only low HGS; the asymmetric-HGS group containing those with only asymmetric HGS; the low-HGS combined with asymmetric-HGS group (BOTH group) with subjects showing both low and asymmetric HGS; the normal group containing subjects with neither low nor asymmetric HGS.

### Muscle mass measurement

In the morning after an overnight fast, participants were required to empty their bladders and bowels before measuring their BIA muscle mass using an InBody770 instrument (BioSpace, Seoul, Korea). The appendicular skeletal muscle mass (ASM) was calculated using the following equation: ASM (kg) = 0.286RI@250 kHz + 1.367sex + 0.054Xc@50 kHz + 0.031body weight − 1.864, where: RI@250 kHz refers to resistance index at 250 kHz derived from BIA; for sex men = 1 and women = 0; Xc@50 kHz refers to reactance at 50 kHz derived from the BIA [18]. This equation was developed using the InBody 770 and was validated based on Asian people. The skeletal muscle mass index (SMI) was calculated as SMI =ASM / height^2^. The definition of low muscle mass, as provided by AWGS2019, is an SMI of < 7.0 kg/m^2^ for males and < 5.7 kg/m^2^ for females [1]. Therefore, for this set of measurements, two groups of subjects could be identified, namely the low muscle mass group containing those who met the criteria for low muscle mass and the non-low muscle mass group that included the remainder of the participants.

### Statistical analysis

SPSS 23.0 software was used for all data analysis. Normally distributed continuous data are presented as mean ± standard deviation (SD), with the median and interquartile range (IQR) used for the non-normally distributed data. Categorical data are shown as value (%). Baseline characteristics between the different groups were compared using t-tests (if the continuous variable followed a normal distribution), the Mann-Whitney U test (if the continuous variable did not follow a normal distribution), and the Pearson chi-square test (for categorical variables). The relationship between muscle mass and different HGS groups was assessed by binary logistic regression. Two models were used in the analysis, namely, an unadjusted model (Model 1) and a model that was adjusted for possible confounding variables (Model 2), i.e., variables for which *P*-values were < 0.05 during univariate analysis.

## Results

Overall, 1748 subjects were enrolled in the study, of whom 1272 (72.77%) were over the age of 60 years. The numbers of Han, Tibetan, and Qiang participants were 885 (50.63%), 217 (12.41%), and 579 (33.12%), respectively. A total of 465 participants (26.60%) were classified into the low muscle mass group, while 228 (13.04%), 536 (30.66%), and 125 (7.15%) were included in the low HGS-only, asymmetric HGS-only, and low HGS combined with asymmetric HGS groups, respectively. There were significant differences in age, marital status, education level, ethnicity, drinking history, smoking history, hypertension history, stroke history, ADL function decreased, gait speed, P-ALB, and ALB between the low muscle mass and the non-low muscle mass groups (Table 1). However, no significant differences were observed in terms of diabetes, CHD, COPD, osteoarthritis, cataracts, and TP between the two groups (Table 1).

**Table 1:** Characteristics of the participants

Characteristics	Non- low muscle mass (*n* = 1283)	low muscle mass (*n* = 465)	T/χ^2^ / Z	*P*-value
**Age**,** years**,** n (%)**			86.55	< 0.001
50–59	399(83.8)	77(16.2)		
60–69	555(77.2)	164(22.8)		
≥ 70	329(59.5)	224(40.5)		
**Ethnic**,** n (%)**			35.219	< 0.001
Han	604(68.2)	281(31.8)		
Tibetan	171(78.8)	46(21.2)		
Qiang	467(80.7)	112(19.3)		
The other	41(62.1)	25(37.9)		
**Education level**,** n (%)**			12.366	0.006
Illiterate	336(71.5)	134(28.5)		
Primary school	424(71.9)	166(28.1)		
Junior high school	327(80.1)	81(19.9)		
Senior high school or above	175(70.3)	74(29.7)		
**Marital status**,** n (%)**			7.558	0.006
Married	1108(74.7)	376(25.3)		
Single/Divorced/Widow	175(66.5)	88(33.5)		
**Smoking history**,** n (%)**			36.289	< 0.001
No	1086(76.5)	333(23.5)		
Yes	173(59.5)	118(40.5)		
**Drinking history**,** n (%)**			5.703	0.017
No	930(75.2)	306(24.8)		
Yes	329(69.6)	144(30.4)		
**Hypertension**,** n (%)**			6.021	0.014
No	830(71.6)	330(28.4)		
Yes	453(77)	135(23)		
**Diabetes**,** n (%)**			1.501	0.221
No	1129(72.9)	419(27.1)		
Yes	154(77)	46(23)		
**CHD**,** n (%)**			0.002	0.96
No	1234(73.4)	447(26.6)		
Yes	49(73.1)	18(26.9)		
**COPD**,** n (%)**			2.649	0.104
No	1199(73.9)	424(26.1)		
Yes	84(67.2)	41(32.8)		
**Stroke history**,** n (%)**			4.516	0.034
No	1240(73.9)	439(26.1)		
Yes	43(62.3)	26(37.7)		
**Osteoarthrosis**,** n (%)**			2.525	0.112
No	902(72.3)	345(27.7)		
Yes	381(76)	120(24)		
**Cataract**,** n (%)**			2.023	0.155
No	1146(73.9)	404(26.1)		
Yes	127(69.2)	61(30.8)		
**ADL function decreased**,** n (%)**			5.336	0.021
No	1196(74.1)	418(25.9)		
Yes	87(64.93)	47(35.07)		
**Gait speed**,** median(iqr)**	1.2(1.1,1.3)	1.1(1,1.3)	-3.829	< 0.001
**TP**,** median(iqr)**	73.9(70.9,77.2)	74.4(71.4,77.5)	-1.492	0.136
**P-ALB**,** median(iqr)**	259(226,291)	251(211, 282)	-3.702	< 0.001
**ALB**,** median(iqr)**	43.9(42.5,45.4)	44(42.5,45.7)	-0.584	< 0.001

Note: CHD: coronary heart disease; COPD: chronic obstructive pulmonary disease; ADL: activities of daily living; TP: total protein; P-ALB: prealbumin; ALB: albumin

**Fig. 1:**
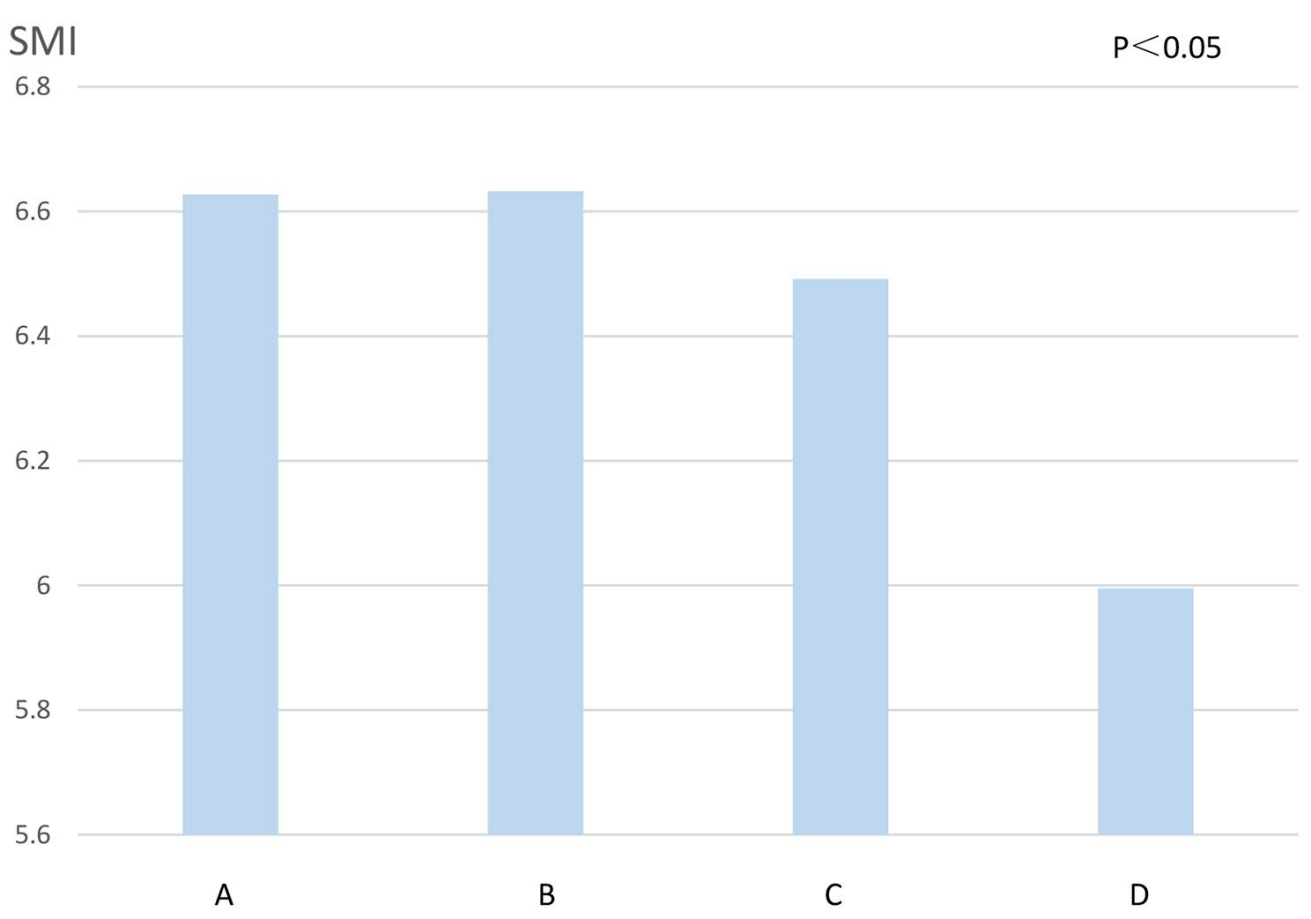
Skeletal muscle index (SMI) of four groups of patients Note: **A**-normal group, the average SMI is 6.627 kg/m^2^; **B**-asymmetrical HGS group, the average SMI is 6.633 kg/m^2^; **C**-low HGS group, the average SMI is 6.492 kg/m^2^; **D**-BOTH, the average SMI is 5.995 kg/m^2^ SMI: skeletal muscle mass index, HGS: handgrip strength

The average SMI values were significantly different among the normal, asymmetric HGS, low HGS, and BOTH groups (normal group vs. asymmetric HGS group vs. low HGS group vs. BOTH group: 6.627 kg/m^2^ vs. 6.633 kg/m^2^ vs. 6.492 kg/m^2^ vs. 5.995 kg/m^2^, respectively, *P* < 0.05, Fig. 1). The prevalence of low muscle mass also increased sequentially in the normal, asymmetric HGS, low HGS, and BOTH groups, with significant differences (normal group vs. asymmetric HGS group vs. low HGS group vs. BOTH group: 21.5% vs. 22.4% vs. 39.5% vs. 56%, respectively, *P* = 0.001, Table 2).

**Table 2:** Univariate analysis of HGS disturbance and low muscle mass

Variable	Non-low muscle mass (*n* = 1283)	low muscle mass (*n* = 465)	*P*
HGS disturbance			0.001
Normal	674(78.5)	185(21.5)	
Only asymmetrical HGS	416(77.6)	120(22.4)	
Only low HGS	138(60.5)	90(39.5)	
BOTH	55(44)	70(56)	

Note: HGS: handgrip strength

Logistic regression analysis further indicated that the risk of developing low muscle mass was higher for subjects in the low HGS (OR = 2.376, 95%CI: 1.74–3.244; Table 3) and BOTH (OR = 4.637, 95%CI: 3.143–6.841; Table 3) groups compared with the normal group. In addition, after adjusting for possible confounding variables, low HGS (OR = 1.7, 95%CI: 1.203–2.402; Table 3) and BOTH (OR = 3.378, 95%CI: 2.173–5.252; Table 3) were found to be independent risk factors for the development of low muscle mass, with the risk being higher in the BOTH group relative to the low HGS group.

**Table 3:** Associations between HGS disturbance and low muscle mass

Variable	Model 1		Model 2	
	*P*-value	OR (95% CI)	*P*-value	OR (95% CI)
HGS disturbance				
Normal	-	1	-	1
Only asymmetrical HGS	0.708	1.051(0.81–1.363)	0.922	1.014(0.767–1.341)
Only low HGS	< 0.001	2.376(1.74–3.244)	0.003	1.7(1.203–2.402)
BOTH	< 0.001	4.637(3.143–6.841)	< 0.001	3.378(2.173–5.252)

Note:

Model 1: a non-adjusted model

Model 2: adjusting for age, sex, ethnic, education level, marital status, smoking history, drinking history, hypertension, stroke history, ADL function decreased, gait speed, P-ALB

HGS: handgrip strength; ADL: activities of daily living; P-ALB: prealbumin

## Discussion

This study showed that low HGS only as well as low HGS combined with asymmetric HGS could predict the chance of low muscle mass, with a higher chance in the latter group. On the other hand, asymmetric HGS alone was not linked to low muscle mass chance. This is the first study to consider the relationship between low muscle mass and different types of HGS. The strength of this study is that we found a relationship between muscle mass and low HGS alone, as well as between low HGS combined with asymmetric HGS, in a community setting. The measurement of muscle mass requires large and expensive instruments, the cost of assessment is relatively high, and measurement requires specialized venues and trained personnel, and there is also the possibility of radiation. However, the equipment used for measuring HGS is relatively cheap and compact, and the cost of assessment is low. The assessments do not require specialized venues and trained personnel, and there are no hidden dangers of radiation. The results of this study thus have clinical utility for the identification of patients with low muscle mass in community settings and institutions not equipped to examine muscle mass itself. Based on the results, it is expected that primary hospitals or other institutions where muscle mass-measuring instruments may not be available, in addition to identifying low HGS as recommended by the AWGS2019, should consider the presence of HGS asymmetry in patients who might have a greater chance of low muscle mass.

The findings show that low HGS can predict the chance of low muscle mass. These results are consistent with earlier studies where a correlation was noted between HGS and muscle mass [8, 9, 19–22]. Muscle strength is influenced by many factors, including the size and length of the muscle fibers that make up the skeletal muscle [23], the muscle structure, and the types of muscle fibers [24]. Hence, it is possible that lower muscle mass may indicate the presence of smaller muscle fibers, which can subsequently lead to less muscle strength. However, the exact mechanism is unclear, and further research would be needed for confirmation.

Asymmetric HGS is a new concept that has been proposed in recent years although no studies have explored the relationship between asymmetric HGS and muscle mass. However, when present alongside low HGS, asymmetric HGS can predict the chance of low muscle mass. Asymmetry in HGS may also suggest deficits in brain function and the neuromuscular system [12, 25]. For instance, studies have shown that HGS asymmetry was a significant predictor of cognitive impairment, as well as a reduced ability to perform physical daily living tasks [13, 25]. Cognitive impairment and a reduced ability to perform physical tasks associated with daily living have been linked to low muscle mass [26, 27]. Therefore, the mechanism through which a combination of low HGS and HGS asymmetry increases the chance of low muscle mass could be related to cognitive impairment and a decreased ability to perform daily living tasks. However, the exact mechanism requires further investigation.

This study was not without limitations. Firstly, no causal relationship between HGS and muscle mass could be established due to the cross-sectional nature of the study. Additional prospective cohort studies would therefore be recommended to confirm this potential relationship. Second, this study was conducted at a fixed location and during a relatively short period of time. Hence, it was unable to include bedridden or hospitalized subjects. It is suggested that future research should expand the research settings by, for example, including community hospitals, nursing homes, or household surveys to apply the findings to a larger population. Third, this study did not include malnutrition, frailty and/or one of the comorbidity indexes. Only indirect and nonspecific indicators of nutritional status such as P-ALB and ALB were included. Finally, the findings of the current study are not applicable to subjects with hand injuries or who were unable to complete the HGS test for other reasons.

## Conclusion

The findings suggest that although asymmetrical HGS itself does not increase the chances of low muscle mass. However, when low HGS and a combination of both features (low HGS combined with asymmetric HGS) is present in subjects, the chance of low muscle mass increases.

## Data Availability

The datasets generated and analyzed during the current study have not been made publicly available because of the large amount of information contained in the databases, which are still under ongoing investigation and analysis, but are now available from the corresponding author upon reasonable request.

## References

[1] Chen L-K, Woo J, Assantachai P, et al. Asian working group for sarcopenia: 2019 consensus update on sarcopenia diagnosis and treatment. J Am Med Dir Assoc. 2020;21(3). 10.1016/j.jamda.2019.12.012. (In eng). 10.1016/j.jamda.2019.12.012 32033882

[2] Xi F, Tan S, Gao T, et al. Low skeletal muscle mass predicts poor clinical outcomes in patients with abdominal trauma. Nutrition. 2021;89:111229. 10.1016/j.nut.2021.111229. (In eng). 33887547 10.1016/j.nut.2021.111229

[3] Xiao J, Caan BJ, Cespedes Feliciano EM, et al. Association of low muscle mass and low muscle radiodensity with morbidity and mortality for colon cancer surgery. JAMA Surg. 2020;155(10):942–9. 10.1001/jamasurg.2020.2497. (In eng). 32805015 10.1001/jamasurg.2020.2497 PMC7424546

[4] Osuna-Padilla IA, Rodríguez-Moguel NC, Rodríguez-Llamazares S, et al. Low muscle mass in COVID-19 critically-ill patients: prognostic significance and surrogate markers for assessment. Clin Nutr. 2022;41(12):2910–7. 10.1016/j.clnu.2022.02.019. (In eng). 35282986 10.1016/j.clnu.2022.02.019 PMC8886683

[5] Wang J, Xu L, Huang S, Hui Q, Shi X, Zhang Q. Low muscle mass and Charlson comorbidity index are risk factors for short-term postoperative prognosis of elderly patients with gastrointestinal tumor: a cross-sectional study. BMC Geriatr. 2021;21(1):730. 10.1186/s12877-021-02683-z. (In eng). 34949161 10.1186/s12877-021-02683-z PMC8705191

[6] Porto JM, Nakaishi APM, Cangussu-Oliveira LM, Freire Júnior RC, Spilla SB, Abreu DCCd. Relationship between grip strength and global muscle strength in community-dwelling older people. Arch Gerontol Geriatr. 2019;82:273–8. 10.1016/j.archger.2019.03.005. (In eng). 30889410 10.1016/j.archger.2019.03.005

[7] Momma H, Kato K, Sawada SS, et al. Physical fitness and dyslipidemia among Japanese: a cohort study from the Niigata wellness study. J Epidemiol. 2021;31(4):287–96. 10.2188/jea.JE20200034. (In eng). 32418939 10.2188/jea.JE20200034 PMC7940973

[8] Bohannon RW. Muscle strength: clinical and prognostic value of hand-grip dynamometry. Curr Opin Clin Nutr Metab Care. 2015;18(5):465–70. 10.1097/MCO.0000000000000202. (In eng). 26147527 10.1097/MCO.0000000000000202

[9] Moreau J, Ordan M-A, Barbe C, et al. Correlation between muscle mass and handgrip strength in digestive cancer patients undergoing chemotherapy. Cancer Med. 2019;8(8):3677–84. 10.1002/cam4.2238. (In eng). 31115188 10.1002/cam4.2238 PMC6639177

[10] Carson RG. Get a grip: individual variations in grip strength are a marker of brain health. Neurobiol Aging. 2018;71:189–222. 10.1016/j.neurobiolaging.2018.07.023. (In eng). 30172220 10.1016/j.neurobiolaging.2018.07.023

[11] McGrath R, Johnson N, Klawitter L, et al. What are the association patterns between handgrip strength and adverse health conditions? A topical review. SAGE Open Med. 2020;8:2050312120910358. 10.1177/2050312120910358. (In eng). 32166029 10.1177/2050312120910358 PMC7052448

[12] McGrath R, Clark BC, Cesari M, Johnson C, Jurivich DA. Handgrip strength asymmetry is associated with future falls in older Americans. Aging Clin Exp Res. 2021;33(9):2461–9. 10.1007/s40520-020-01757-z. (In eng). 33247424 10.1007/s40520-020-01757-z PMC8211412

[13] Parker K, Rhee Y, Tomkinson GR, Vincent BM, O’Connor ML, McGrath R. Handgrip weakness and asymmetry independently predict the development of new activity limitations: results from analyses of longitudinal data from the US health and retirement study. J Am Med Dir Assoc. 2021;22(4). 10.1016/j.jamda.2020.11.006. (In eng). 10.1016/j.jamda.2020.11.006 33290729

[14] McGrath R, Tomkinson GR, LaRoche DP, Vincent BM, Bond CW, Hackney KJ. Handgrip strength asymmetry and weakness may accelerate time to mortality in aging Americans. J Am Med Dir Assoc. 2020;21(12). 10.1016/j.jamda.2020.04.030. In eng. 10.1016/j.jamda.2020.04.030 32611522

[15] Klawitter L, Vincent BM, Choi B-J, et al. Handgrip strength asymmetry and weakness are associated with future morbidity accumulation in Americans. J Strength Cond Res. 2022;36(1):106–12. 10.1519/JSC.0000000000004166. (In eng). 34941610 10.1519/JSC.0000000000004166

[16] Luo S, Chen X, Hou L, et al. The relationship between sarcopenia and vitamin D levels in adults of different ethnicities: findings from the West China health and aging trend study. J Nutr Health Aging. 2021;25(7):909–13. 10.1007/s12603-021-1645-z. (In eng).34409970 10.1007/s12603-021-1645-z

[17] Katz S, Ford AB, Moskowitz RW, Jackson BA, Jaffe MW. Studies of illness in the aged. The index of adl: a standardized measure of biological and psychosocial function. JAMA. 1963;185:914–9. 10.1001/jama.1963.03060120024016 14044222 10.1001/jama.1963.03060120024016

[18] Zhou J, Luo L, Xie L, et al. Sarcopenic obesity by the ESPEN/EASO criteria for predicting mortality in advanced non-small cell lung cancer. Clin Nutr. 2023;42(6):817–24. 10.1016/j.clnu.2023.04.010. (In eng). 37084468 10.1016/j.clnu.2023.04.010

[19] Campbell TM, Vallis LA. Predicting fat-free mass index and sarcopenia in assisted-living older adults. Age (Dordr). 2014;36(4):9674. 10.1007/s11357-014-9674-8. (In eng). 24994536 10.1007/s11357-014-9674-8 PMC4150904

[20] Jiang K, Singh Maharjan SR, Slee A, Davenport A. Differences between anthropometric and bioimpedance measurements of muscle mass in the arm and hand grip and pinch strength in patients with chronic kidney disease. Clin Nutr. 2021;40(1):320–3. 10.1016/j.clnu.2020.04.026. (In eng). 32414538 10.1016/j.clnu.2020.04.026

[21] Fülster S, Tacke M, Sandek A, et al. Muscle wasting in patients with chronic heart failure: results from the studies investigating co-morbidities aggravating heart failure (SICA-HF). Eur Heart J. 2013;34(7):512–9. 10.1093/eurheartj/ehs381. (In eng). 23178647 10.1093/eurheartj/ehs381

[22] Itoh S, Shirabe K, Yoshizumi T, et al. Skeletal muscle mass assessed by computed tomography correlates to muscle strength and physical performance at a liver-related hospital experience. Hepatol Res. 2016;46(4):292–7. 10.1111/hepr.12537. (In eng). 26031324 10.1111/hepr.12537

[23] Odegard GM, Donahue TLH, Morrow DA, Kaufman KR. Constitutive modeling of skeletal muscle tissue with an explicit strain-energy function. J Biomech Eng. 2008;130(6):061017. 10.1115/1.3002766. (In eng). 19045546 10.1115/1.3002766 PMC2823080

[24] Fitts RH, McDonald KS, Schluter JM. The determinants of skeletal muscle force and power: their adaptability with changes in activity pattern. J Biomech. 1991;24(Suppl 1):111–22. 10.1016/0021-9290(91)90382-w. (In eng). 1791172 10.1016/0021-9290(91)90382-w

[25] Choi J-Y, Lee S, Min J-Y, Min K-B. Asymmetrical handgrip strength is associated with lower cognitive performance in the elderly. J Clin Med. 2022;11(10). 10.3390/jcm11102904. (In eng). 10.3390/jcm11102904 PMC9144314 35629029

[26] Wang DXM, Yao J, Zirek Y, Reijnierse EM, Maier AB. Muscle mass, strength, and physical performance predicting activities of daily living: a meta-analysis. J Cachexia Sarcopenia Muscle. 2020;11(1). 10.1002/jcsm.12502. (In eng). 10.1002/jcsm.12502 PMC7015244 31788969

[27] Oudbier SJ, Goh J, Looijaard SMLM, Reijnierse EM, Meskers CGM, Maier AB. Pathophysiological mechanisms explaining the association between low skeletal muscle mass and cognitive function. J Gerontol Biol Sci Med Sci. 2022;77(10):1959–68. 10.1093/gerona/glac121. (In eng). 10.1093/gerona/glac121 PMC9536455 35661882

